# StingReady: A Novel Device for Controlled Insect Sting Challenge—From Field Capture to Clinical Application

**DOI:** 10.3390/toxins17060260

**Published:** 2025-05-22

**Authors:** Xesús Feás, Margarita Armisén, Sara López-Freire, Manuela Alonso-Sampedro, Carmen Vidal

**Affiliations:** 1 Academy of Veterinary Sciences of Galicia, Edificio Escola Galega de Administración Pública, Rúa Madrid, No. 2–4, 15707 Santiago de Compostela, Spain; xesusfeas@gmail.com; 2 Fundación Instituto de Investigación Sanitaria de Santiago de Compostela (FIDIS), Hospital Clínico, 15706 Santiago de Compostela, Spain; manuela.alonso.sampedro@sergas.es; 3 Allergy Department, University Hospital of Santiago de Compostela, 15706 Santiago de Compostela, Spain; margarita.armisen.gil@sergas.es (M.A.); saralopzfreire@gmail.com (S.L.-F.); 4 Research Methods Group (RESMET), Health Research Institute of Santiago de Compostela (IDIS), University Hospital of Santiago de Compostela, 15706 Santiago de Compostela, Spain; 5 Network for Research on Chronicity, Primary Care, and Health Promotion (RICAPPS-ISCIII/RD21/0016/0022), University Hospital of Santiago de Compostela, 15706 Santiago de Compostela, Spain; 6 Department of Psychiatry, Radiology, Public Health, Nursing and Medicine, Faculty of Medicine, University of Santiago de Compostela (USC), 15782 Santiago de Compostela, Spain

**Keywords:** venom immunotherapy, sting challenge, *Vespa velutina*, hymenoptera, allergy testing, insect handling, medical device

## Abstract

Reliable assessment of protection in venom immunotherapy (VIT) patients remains a clinical challenge, especially due to the limitations of conventional sting challenge tests (SCTs), which require complex insect handling and may compromise test accuracy. This study introduces StingReady, a novel, user-friendly device designed to streamline the SCT process by enabling safe, efficient, and minimally manipulative exposure to hymenopteran stings. For the first time, StingReady was applied to conduct SCTs with *Vespa velutina*, an invasive hornet species of increasing clinical relevance. The device was tested in a real-world setting at Belvís Park in Santiago de Compostela, Spain, where hornets were successfully captured and transported to the hospital without anesthesia or limb removal. The design features adjustable mesh sizes, allowing compatibility with various hymenopteran taxa. Using StingReady, nine patients underwent SCTs with no need for direct insect handling during the hospital procedure. The process improved patient safety and comfort while preserving the insect’s natural stinging behavior, thereby enhancing test reliability. This study demonstrates that StingReady significantly improves SCT methodology, offering a practical, reproducible, and ethically sound alternative for evaluating VIT efficacy across diverse hymenopteran species.

## 1. Introduction

Hymenoptera venom allergy is a serious medical condition that can lead to severe allergic reactions and, in some cases, death following stings from venomous insects such as bees, wasps, and hornets [1]. Effective diagnostic and treatment strategies are essential for managing this potentially life-threatening condition, especially in patients with venom hypersensitivity. Venom immunotherapy (VIT) is the cornerstone treatment for individuals with a history of severe allergic reactions to hymenoptera stings, as it significantly reduces the risk of future systemic reactions [2,3,4]. VIT gradually induces changes in the immune system of patients allergic to the venom due to controlled administration to repeated doses of the allergen. This approach also offers long-term protection, enhancing the quality of life for patients with venom hypersensitivity [5].

The sting challenge test (SCT) with hymenoptera venom involves the provocation of a real sting with a live insect in an allergic patient as an essential tool for assessing the efficacy of VIT [6,7,8]. The SCT tries to simulate an in-field sting and determine whether a patient has achieved immune tolerance through VIT, which is crucial for guiding treatment adjustments or possible discontinuation [9,10,11,12]. In addition, as has been demonstrated, the experience of a well-tolerated sting exposure has been shown to result in improvements in quality of life because of the alleviation of patients’ fear and anxiety associated with stings [13,14,15].

Despite its clinical benefits, the SCT is underused due to the logistical challenges of sourcing and handling live insects in clinical environments [16,17,18,19,20]. Traditionally, the SCT approach involves the anesthetization of insects and the removal of wings and legs to facilitate application on the patient’s skin. This method not only increases the risk of venom loss but also requires significant expertise to avoid injuring the insect, which could result in incomplete venom delivery [21]. Finally, the need for precise manipulation with specialized tools adds complexity, making the procedure less accessible for practitioners without entomological training.

In response to the complexities of conventional SCT methods, we have tried to simplify the procedure by designing a versatile device compatible with various hymenopteran species and adaptable to different insect sizes. While the device is designed for broad utility, our primary focus was *Vespa velutina*, an invasive species spreading rapidly across Europe [22]. *V. velutina* has become a significant problem for beekeepers and rural dwellers alike. This species of hornet is known to attack honey bees, causing damage to their colonies, and its sting can lead to severe allergic reactions in humans [23]. Our group have studied the allergenic and toxicological impact of *V*. *velutina* and identified Vesp v 5 and glycosylated Vesp v 1 as relevant allergens [24]. Tests, such as specific IgE and the basophil activation test, show consistency in *V*. *velutina* allergies [25], while preliminary evidence suggests immunotherapy with *Vespula* venom as a potential treatment [23]. Environmental factors and nest architectures have also been examined [22], along with studies focusing on venom protocols [21]. Fatalities and sting epidemiology, particularly in Spain, have also been documented by Feás, offering insights into the broader impacts of stinging insects in Europe [1,26].

In this study, we introduce the StingReady device and demonstrate its practical application for conducting sting challenges in a clinical setting. For the first time, we performed controlled SCTs using *V*. *velutina* on a cohort of nine patients, providing insights into the device’s efficacy and the potential to enhance allergy management protocols for hymenoptera venom allergy cases. This innovation could enable more widespread, standardized, and accurate assessments of VIT effectiveness, ultimately improving patient outcomes.

## 2. Results

### 2.1. Capture of Vespa velutina Specimens

**Figure 1:**
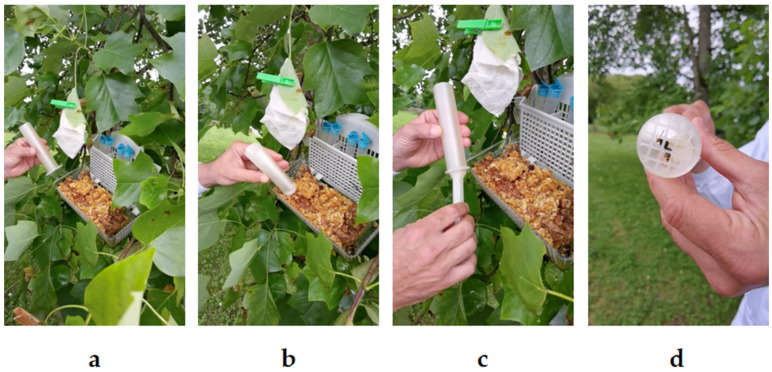
Sequential capture process of *Vespa velutina* using the StingReady device in the field. (**a**) The hornet feeds at the bait station as the containment chamber (CCh) is carefully positioned nearby. (**b**) The chamber encloses the insect, encouraging it to move upwards toward the mesh area. (**c**) The gliding component (GC) is inserted with both hands to fully secure the hornet. (**d**) The *V. velutina* is now safely confined within the StingReady device, which is held securely in one hand. Photo author: A. Martín.

The capture of *V. velutina* was conducted in pre-identified hotspots within Belvís Park (Figure 1). These locations were chosen based on the seasonal blooming of specific plants known to attract hymenopteran insects in general, and *V. velutina* particularly. Bait stations were also strategically placed near the flowering plants to enhance the attraction of hornets. The process yielded successful captures, ensuring that enough hornets were available for the SCTs. The *V. velutina* specimens were captured the day before the SCT and maintained under controlled conditions until the test (darkness and fed ad libitum) ensuring that the insects remained healthy and ready for the SCT.

**Figure 2:**
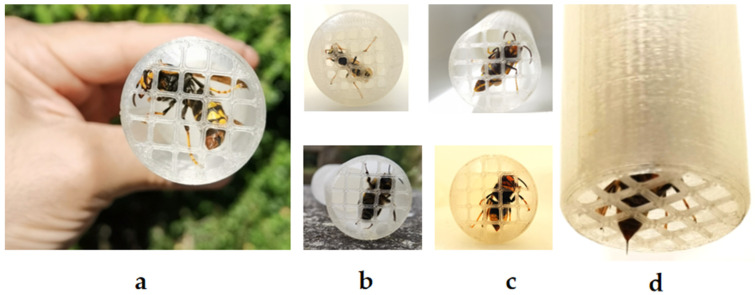
Close-up views of insect species captured within the StingReady device. (**a**) A *Vespa velutina* queen (gyne) in containment. (**b**) Top: *Polistes* spp. with a modified chamber mesh size of 3 mm; bottom: *Bombus* spp. specimen. (**c**) Top: *Vespa velutina* male; bottom: *Vespa velutina* worker. (**d**) Detail of the mesh showing the insect’s stinger and part of the abdomen extending through, as seen with a *Vespa velutina worker*, allowing safe sting access to the skin. Photo author: X. Feás.

The close-up images of various insect species within the StingReady device highlight the mesh’s dual role in secure containment and in enabling feeding and controlled sting delivery (Figure 2). The mesh size is specifically designed to allow the insect’s stinger and part of the abdomen to access the skin, as shown in the image of a *V. velutina* queen positioned for sting induction. While *Bombus* spp. and *Polistes* spp. were not primary targets, their successful capture further underscores the adaptability of the mesh for smaller species. Male hornets were also easily identified and excluded from capture. However, a male *V*. *velutina* was positioned in the device to illustrate morphological distinctions.

### 2.2. StingReady Device Performance and Observation of Sting Reactions

**Figure 3:**
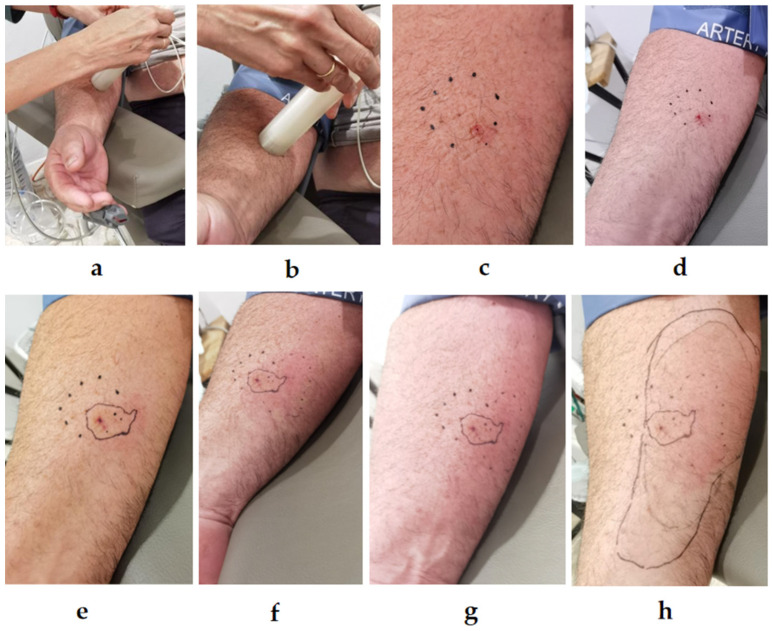
Procedure for applying a controlled sting using the StingReady device. (**a**) Initial positioning of the device on the inner forearm over the flexor muscles, with the mesh in contact with the skin. (**b**) The *Vespa velutina* is carefully guided into position for stinging by lowering the gliding component. (**c**) Close-up immediately after the sting, showing the characteristic red puncture mark from the stinger (Time 0). (**d**) 15 min after the sting, (**e**) 31 min, (**f**) 46 min, (**g**) 71 min, and (**h**) 130 min post-sting. Photo author: X. Feás.

**Figure 4:**
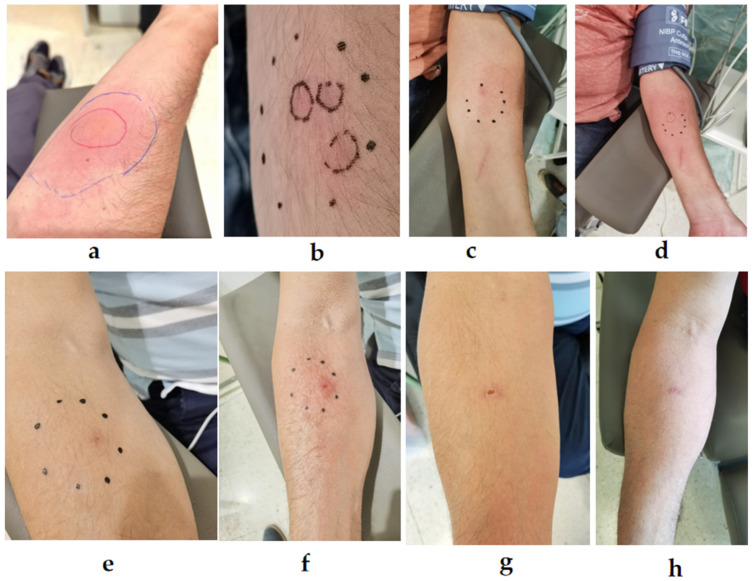
Progression of sting reactions from *Vespa velutina* stings in different patients. (**a**) Patient with localized reaction immediately after a single sting. (**b**) Patient with three distinct stings. (**c**) Initial sting reaction in a patient directly post-sting, and (**d**) after 1 h. (**e**) Immediate reaction in a different patient, (**f**) 8 min post-sting, (**g**) 12 days post-sting, and (**h**) 19 days post-sting. Photo author: X. Feás.

The StingReady device was used for the sting challenge tests with nine patients. Following the sting application, the patients’ skin reactions were observed and documented. Images were taken of the sting sites, and the inflammatory responses were evaluated (Table 1, Figure 3 and Figure 4). In one instance, a *V. velutina* successfully stung the patient three times, demonstrating the capability of wasps and hornets to sting multiple times (Figure 4b), in contrast to bees, which lose their stinger after a single sting. The application of the StingReady device ensured that the venom was fully delivered in each instance, with no complications in handling.

## 3. Discussion

The StingReady device demonstrated significant advantages in performing sting challenges, particularly when compared to traditional methods [20]. Conventional techniques for sting challenges involve anesthetizing insects, removing their wings and legs, and handling them with specialized tools like forceps to ensure a controlled sting. These methods not only present challenges in preserving the insect’s integrity but also pose risks of incomplete venom delivery due to mishandling or venom loss during the preparation process. By contrast, the StingReady device eliminates the need for anesthesia and physical alterations to the insects, allowing for a more ethical, efficient, and reliable method of conducting sting challenges. The use of the device also reduces the skill level required by practitioners, making the procedure more accessible. By addressing the primary drawbacks of traditional methods—complexity, risk, and ethical concerns—the StingReady offers a streamlined, safe, and ethical alternative. Although invertebrates, including insects, are not currently subject to animal welfare laws in most jurisdictions, the growing recognition of potential sentience in some invertebrates has prompted ethical considerations within the scientific community. The New York Declaration emphasizes that absolute certainty about consciousness is not required to extend moral consideration to animals. This perspective suggests an obligation to minimize harm even to organisms traditionally excluded from welfare legislation. While the SCT is not constrained by legal frameworks for invertebrate welfare, its ability to reduce risk and harm aligns with emerging ethical standards in scientific practice [27].

The process of collecting hymenopteran specimens remains a logistical challenge for medical professionals, particularly given the difficulties in obtaining live insects. While honey bees and bumble bees can be sourced from beekeepers and commercial breeders, vespids are not as easily available. *V. velutina*, however, is now widespread in Galicia, and its capture can be facilitated by understanding the insect’s behavior and preferred environments. Our study identified four key locations within Belvís Park in Santiago de Compostela for capturing these hornets, selected based on the seasonal blooming of specific plants that attract them. Additionally, bait stations were employed to increase the attraction of *V. velutina* to these areas, with attractants to further enhance capture success. These attractants were selected based on their effectiveness in luring *V. velutina* and other hymenopteran insects [21]. *V. velutina* is not only common in urban environments, where it uses human-made structures for nesting but is also a serious threat to honey bee populations, particularly in apiaries. At apiaries, *V*. *velutina* hunts honey bees by hovering near hive entrances and intercepting foraging bees. This behavior makes apiaries also ideal locations for capturing the hornets, especially during late summer and autumn when their populations peak [22]. In addition to apiaries, other fruitful areas for collecting *V*. *velutina* include orchards and vineyards, where the hornets are attracted to ripe fruit due to their preference for sugary substances. This versatility in capture locations highlights the potential for widespread use of the StingReady device in various environments, providing flexibility for practitioners in different regions.

Once captured, the hornets were confined within the StingReady device and maintained in darkness until the sting challenge. The insects were fed ad libitum using syringes inserted through the mesh containment system of the device, ensuring they remained healthy and viable for the procedure. Although not an objective of the present study, it is worth noting that the insects not used for the SCT were successfully kept in captivity for up to 10 days without any apparent issues. This suggests that the device not only allows for maintaining the insects in good condition but also offers the possibility of safely transporting them for SCT conducted at different locations. The StingReady device’s ability to capture and maintain *V*. *velutina* without causing harm to the insect or compromising the venom delivery during the SCT was further supported by field data.

We present here the results of StingReady applied to nine allergic patients to identify the protection achieved with venom immunotherapy. In one of the patients, the captured *V*. *velutina* was able to sting the patient three times, illustrating a key difference between vespid and bee stings. Unlike bees, which insert their barbed stinger into the skin and die as a result of the stinger being ripped from their body, vespids like *V*. *velutina* have smooth stingers that allow them to sting multiple times without injuring themselves. This distinction is important in the context of sting challenges, as the ability of vespids to sting more than once can lead to multiple venom exposures during the procedure, increasing the efficacy of the test. Although this may initially raise concerns about dose standardization, the primary clinical goal is not to control the exact number of stings but to ensure that venom is injected under controlled and medically supervised conditions. In this regard, the use of StingReady facilitates the real-time observation of the sting and allows healthcare professionals to confirm that envenomation has occurred safely and effectively.

The images included in the Results section offer visual confirmation of the practical utility of the StingReady device. From the field capture of *V. velutina* hornets to their application in the clinic, the device proved to be a reliable tool that simplifies the complex procedures involved in SCT. This represents a major improvement over previous techniques, which often required specialized entomological expertise and equipment to anesthetize or handle the insects. Additionally, this method eliminates the need to mutilate or harm the insects, as they are neither anesthetized nor immobilized, allowing for a more humane and efficient process.

The practical implications of the StingReady device are substantial. First, it simplifies the process of obtaining and utilizing insects for sting challenges, making it more accessible to clinical settings that may lack entomological expertise or specialized equipment. The ease of capture, transport, and application reduces the need for extensive training or the presence of entomologists during the procedure, thereby lowering operational costs and time.

From a clinical point of view, the fact that the application on the skin can be performed without prior preparation of the insects facilitates the work of the clinician who only has to focus on direct patient care. Although most of the procedures have been performed in the critical care unit, it did not pose any problems or risks in the unit because inside StingReady, it is not possible for the insect to escape and harm other patients or staff. With the exception of patient #4, all patients exhibited a satisfactory tolerance to the sting, thereby substantiating the efficacy of *Vespula* spp. venom in treating the aforementioned patients. Patient #4 underwent the sting challenge shortly after commencing VIT. This positive result consequently prompted a modification in VIT, thus resulting in the incorporation of *V*. *velutina* venom into the treatment regimen. Consequently, the patient is currently undergoing the administration of both vaccines.

Moreover, the StingReady device’s adaptability for different insect species is a key innovation. The ability to adjust the mesh size of the CCh allows it to be used with a wide range of hymenopterans, from smaller bees to larger hornets, potentially broadening its use in venom immunotherapy across different geographic regions and clinical needs. While this study focused on *V. velutina*, the potential applications of the StingReady device extend to other medically relevant insect species. Future research should explore the device’s efficacy with various species, including honey bees, bumble bees, and other wasps. Another avenue for future research is the application of the StingReady device in ecological studies, where live insect handling is required. Its robust design could facilitate more efficient and humane insect handling in these contexts, further expanding its utility beyond clinical environments.

Overall, the introduction of the StingReady device could lead to a more standardized and accessible approach to conducting sting challenge tests, making this critical diagnostic procedure more widely available to allergists. The elimination of many of the complexities involved in handling *V. velutina* and other hymenoptera species could help facilitate more accurate assessments of VIT efficacy, ultimately improving patient outcomes and expanding the clinical application of this therapy. The ability to use the SCD directly from field capture to clinical application without additional equipment or handling makes it an invaluable tool for allergists and immunologists.

## 4. Conclusions

The StingReady device offers a comprehensive solution for conducting SCTs, from insect capture in the field to safe and effective venom delivery in clinical settings. Its design facilitates the direct capture of insects, such as *V. velutina*, eliminating the need for additional tools like nets. The device’s Cch ensures that insects remain confined and viable until use, with an easy method for ad libitum feeding. Furthermore, the StingReady device can be adapted for use with different hymenopteran species—such as bees, bumble bees, and smaller wasps—by modifying the mesh size. We have successfully tested resized versions of the device with *Polistes* spp., *Vespula* spp., *Bombus* spp., and *Apis mellifera*, confirming that these insects can sting through the mesh in a controlled and reproducible manner. This innovation provides a safer, more efficient, and standardized approach to VIT evaluation, with the potential to become a valuable tool in clinical practice.

## 5. Materials and Methods

### 5.1. StingReady

**Figure 5:**
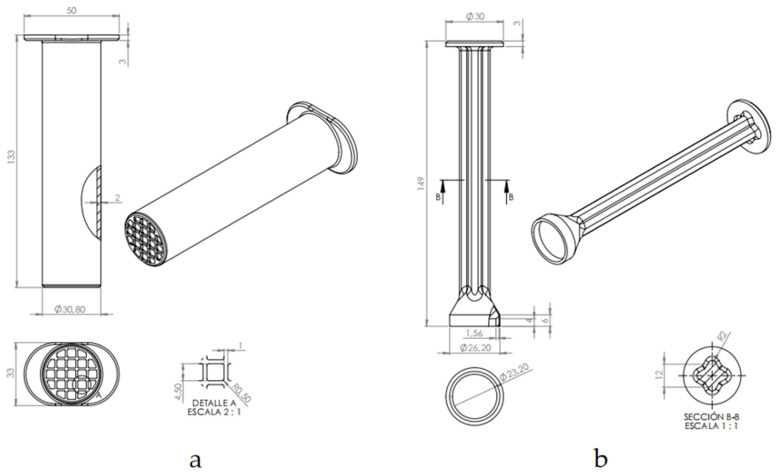
Cross-sectional and perspective views of the StingReady device: (**a**) the collection chamber (Cch), illustrating the device dimensions and detailed structure, including a close-up of the mesh securing feature; (**b**) side and perspective views of the gliding component (GC), highlighting the containment area at the terminal end designed to safely hold the insect in place for a controlled sting.

The StingReady device (Figure 5) is a 3D-printed tool specifically designed for capturing and safely handling hymenoptera insects, such as *Vespa velutina*, for use in clinical settings. The device consists of two main components: a containment chamber (CCh) and a gliding component (GC) that fit together to confine the insect while allowing controlled interaction.

The containment chamber (CCh) (Figure 5a) is a translucent cylindrical structure with a rigid mesh end. This mesh allows safe interaction with the insect without risk of escape while ensuring that the stinger can be exposed to the patient’s skin for use in SCT. The material’s translucency allows monitoring of the insect and ensures its well-being during transport and handling.

The gliding component (GC) (Figure 5b) is designed to be moved within the CCh, in a gently way, guiding the insect towards the mesh end without causing harm. This component includes a protective cap that securely encloses the insect, holding it in place at the mesh end. This design ensures that the insect remains contained and positioned for safe, controlled use in clinical procedures.

### 5.2. Study Area and Temporal Framework

Field research aimed at collecting the *V. velutina* insects was conducted in 2023 and 2024 at various locations within Belvís Park, situated in the historic city center of Santiago de Compostela (NW Spain). This park covers an area of 30,604 m^2^, encompassing former orchards transformed into an urban green space with expansive lawns, groves with large trees, and small gardens maintained by local residents.

### 5.3. Insect Collection Points

**Figure 6:**
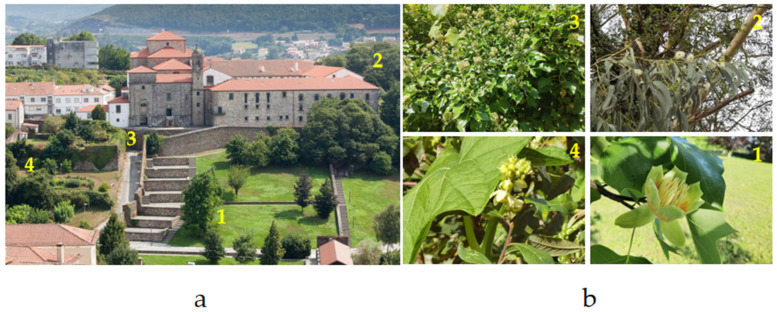
Collection points for *Vespa velutina* and other hymenopterans in Belvís Park. The left side of the figure (**a**) shows part of Parque de Belvís with numbered collection sites (1–4) for insect sampling. The right side (**b**) displays images of the specific flowering plants associated with each point: (1) *Liriodendron tulipifera* (tulip tree), (2) *Eucalyptus* spp. (eucalyptus), (3) *Hedera helix* (ivy), and (4) *Sechium edule* (chayote). Each site corresponds to periods of plant flowering which attract hymenopterans. Photo Author: X. Feás.

Four specific collection points within Belvís Park were selected based on the presence of flowering plants known to attract *V*. *velutina* at different times of the year. Specifically, monitoring and capture efforts were scheduled to align with the nectaring periods of the following plant species within the park: (i) *Liriodendron tulipifera* (tulip tree), April to May; (ii) *Sechium edule* (chayote), August to October; (iii) *Hedera helix* (common ivy), September to November; and (iv) *Eucalyptus* sp., November to December (Figure 6).

**Figure 7:**
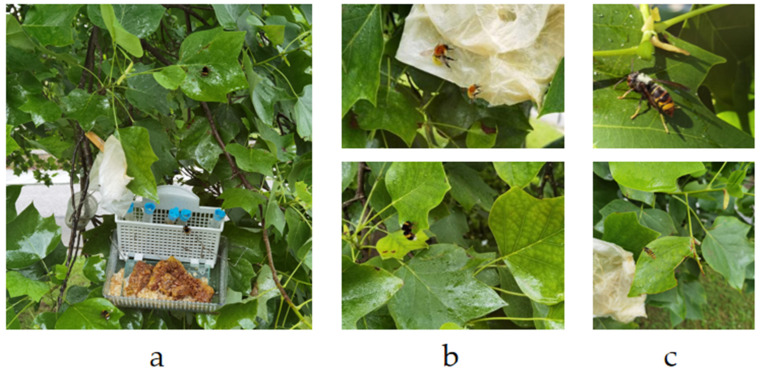
Details of bait station and insect visitation. (**a**) Close-up of the bait station, hung 1 m above ground from *Liriodendron tulipifera* (tulip tree) branches. (**b**) Images of several *Bombus* spp. (bumblebee) visiting the station. (**c**) *Vespa velutina* (top) and *Vespula* spp. (bottom) observed at the bait, illustrating the range of hymenopteran species attracted to the station. Photo Author: X. Feás.

In each of these locations, bait stations (Figure 7) were established to enhance the attraction of *V*. *velutina*. The bait consisted of a combination of bee wax, honey remnants, and homemade attractant solutions made from fermented sugar, water, and baker’s yeast. The attractants used in the bait stations were regularly replenished to ensure continued effectiveness. Next to each bait station, absorbent paper strips are placed to enhance insect attraction. These strips are treated with 10 mL of a 1% geraniol (Sigma-Aldrich, Tres Cantos, Spain) solution in water, which serves as an additional attractant to lure hymenopteran to the area.

### 5.4. Identification, Caste and Sex Determination of Vespa velutina Individuals

**Figure 8:**
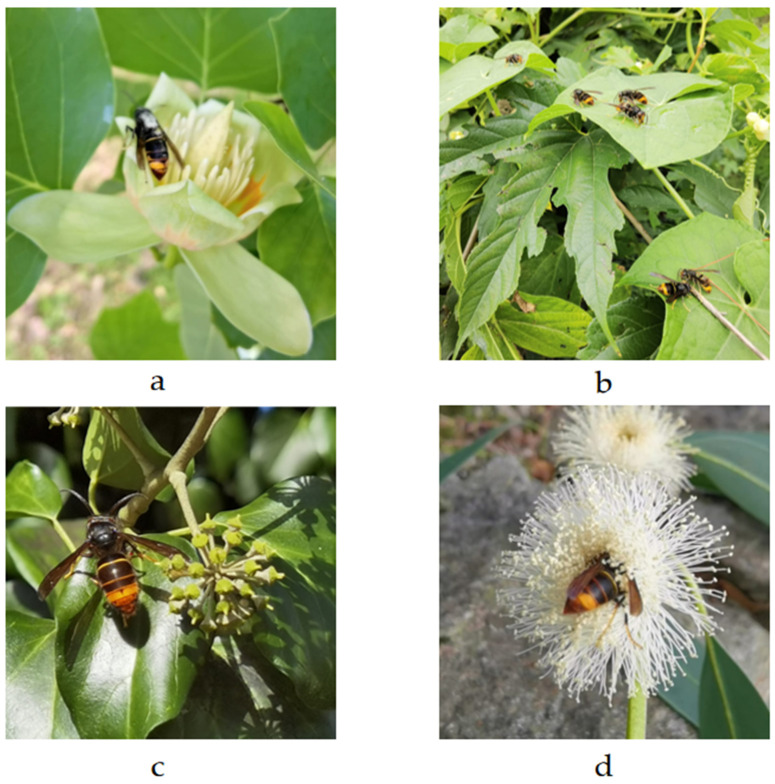
*Vespa velutina* specimens observed on four different flowering plant species within Belvís Park, each corresponding to a specific stage or caste of the species. (**a**) Founder queen on *Liriodendron tulipifera* (tulip tree); (**b**) workers on *Sechium edule* (chayote); (**c**) male on *Hedera helix* (common ivy); and (**d**) gyne on *Eucalyptus* sp. Photo Author: X. Feás.

The insects were identified and sexed based on their external morphological features (Figure 8). Specifically, *V. velutina* typically measures between 2 and 3 cm in length. It has a black head, with an orange face and mouthparts, and antennae that are brown on the dorsal side and orange on the ventral side. The thorax is a dark brown, almost black, color. The metasomal terga are brown, featuring a thin yellow band on the first segment and thin orange bands on the second and third segments, while the fourth metasomal segment is orange, and segments five and six are orange-brown. The legs are brown with yellow tarsi, and the wings have a brownish-hyaline appearance. Female and male hornets exhibit sexual dimorphism. Female hornets have 12 antennal segments (including the scape and pedicel), while male hornets have relatively long, curly-ended antennae with 13 segments (Figure 8c). Female hornets have 6 gastral segments and male hornets have 7 exposed gastral segments. Males have a bilobate apex of the last sternite, which appears sharp in females. The difference between workers (Figure 8b) and reproductive females, queen (Figure 8a) and gynes (Figure 8d), i.e., future queens, was established at a mesoscutum width of 4.5 mm [22].

### 5.5. Capture of Vespa velutina

**Figure 9:**
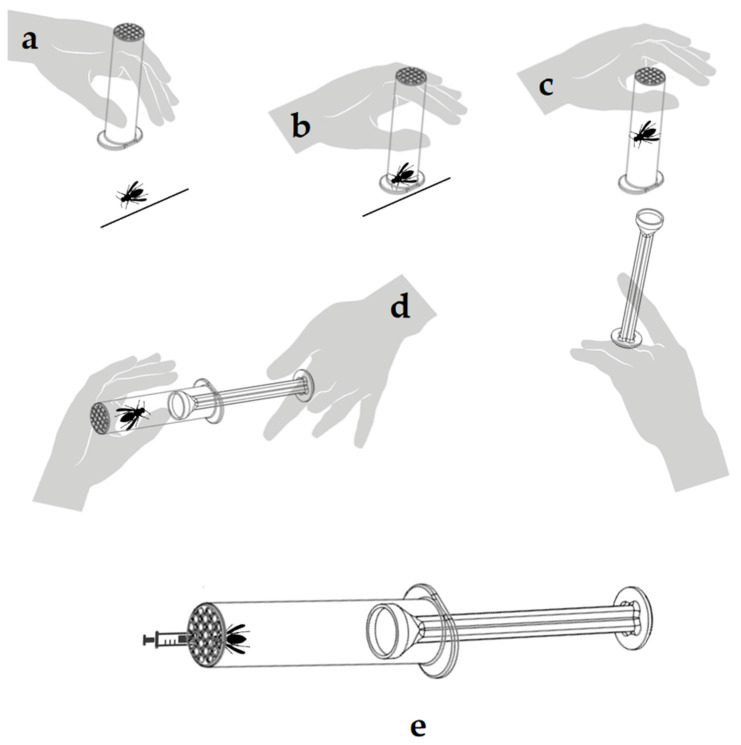
Step-by-step capture of a *Vespa velutina* specimen using the StingReady device. (**a**) The hornet feeding at a bait station. (**b**) The device enclosing the hornet. (**c**) The GC being deployed to confine the insect. (**d**) The hornet securely confined within the StingReady device. (**e**) Feeding of the captured hornet using a 2.5 mL syringe inserted through the mesh, providing a honey–water solution (20%) to maintain hydration and energy levels.

The capture process of *V*. *velutina* was conducted at one of our four designated collection points. To capture the hornet, the CCh of the StingReady device is slowly brought close to the insect while it remains occupied at the bait station (Figure 9a). Once the chamber is properly positioned, it is used to swiftly enclose the hornet, allowing it to remain calm and naturally move upward towards the mesh end of the chamber (Figure 9b). In the next step, the GC is gently introduced into the chamber while both hands stabilize the device, ensuring the hornet is securely confined without harm (Figure 9c). Finally, the complete StingReady device is held securely, with the hornet safely contained within, ready for transport (Figure 9d). A video supplement demonstrating the entire capture process is included in this study to provide a detailed visual guide for replicating the methodology (Supplementary Video S1). After capture, the insects were kept in darkness until the time of the SCT to minimize stress and activity. During this period, they were fed ad libitum using one 2.5 mL syringe filled with a honey–water solution (80% honey, 20% water) (Figure 9e). The tip of the syringe was carefully inserted through the mesh of the Cch, allowing the insect to access food without being disturbed.

### 5.6. Clinical Application of StingReady

The Sting Challenge Test (SCT) was conducted at the Allergy Service of the Complejo Hospitalario Universitario de Santiago de Compostela in nine patients (eight males and one female), aged between 25 and 69 years, with a documented history of severe allergic reactions to *V. velutina* stings. All patients were being treated with *Vespula* spp. venom (Alutard SQ, ALK, Denmark) 100 µg every 8 weeks after confirming IgE sensitization against *Vespula* venom components. *Vespula* spp venom was chosen for immunotherapy because of the lack of previous stings due to *V. velutina* in most of the patients. As for patients #4 and 5, *Vespula* spp was selected because of the highest level of sIgE against *Vespula* spp with respect to *V. velutina*. The median duration of VIT prior to the SCT was 50 months, with a range between 7 and 63 months. Detailed patient demographics, the implicated insect species, and VIT information are summarized in Table 2. SCTs were conducted under controlled clinical conditions, with the continuous monitoring of vital signs and the immediate availability of emergency treatments to address potential adverse reactions.

**Figure 10:**
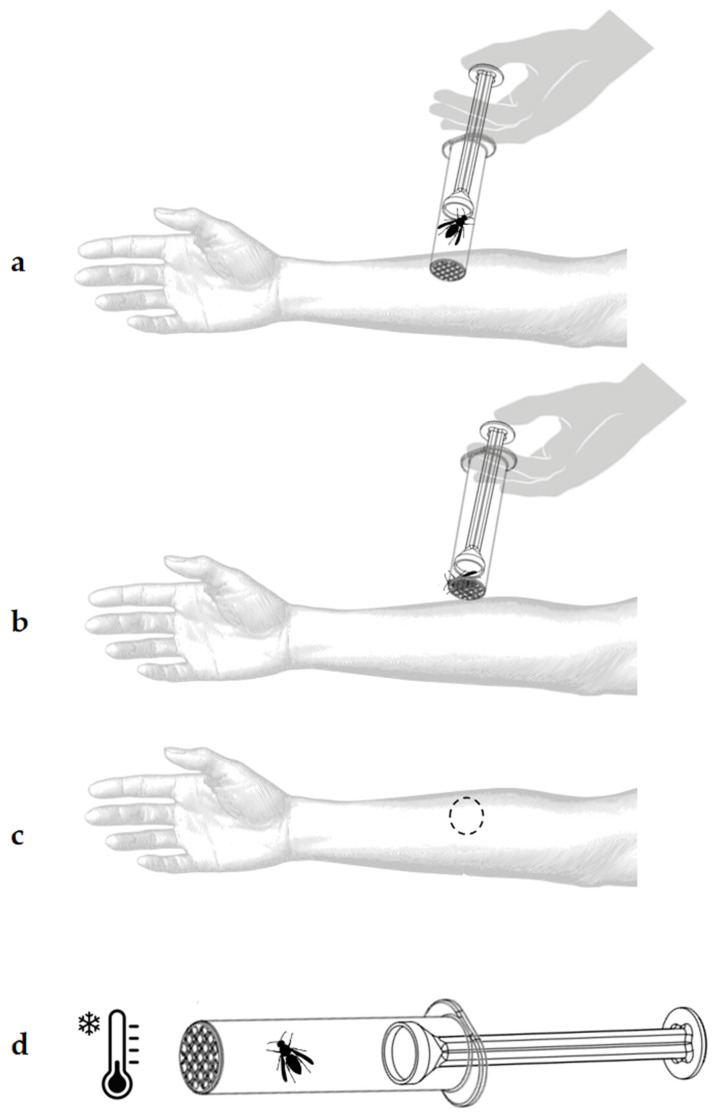
Procedure for using the StingReady device to induce a controlled sting. (**a**) The initial placement of the device in contact with the patient’s skin. (**b**) The activation of the gliding mechanism to position the insect for stinging; the device is held in place for 15 s after the initial sting occurs. (**c**) Marking the area after removing the device to document the sting location. (**d**) Euthanasia of the insect by placing the device in a freezer at −18 °C for five minutes.

Upon the patient’s arrival at the clinic, the captured *V. velutina* is prepared for the SCT (Figure 10) to assess the effectiveness of VIT. The process is as follows:

- Preparation and explanation: The procedure is reviewed with the patient, who is positioned comfortably in a bed at the critical care unit of the hospital. After reading and asking any questions about the procedure, the patient signs the informed consent form. The patient is, then, monitored and with an accessible peripheral venous line.
- Application on to the forearm: The open end of the CCh, where the mesh is located, is placed in direct contact with the patient’s skin on the volar surface of the forearm (Figure 10b). The GC is then gently advanced to position the hornet near the mesh. With light pressure, the insect is encouraged to sting.
- Observation of the sting reaction: Once the patient feels the sting, the hornet is kept in position, confined against the mesh, for, at least, 15 s to ensure a complete sting. The skin reaction is marked and closely observed, and any immediate signs of allergic response are noted (Figure 10c).
- Euthanasia of the insect: After the procedure, the hornet is euthanized by placing the StingReady device in a freezer at −18 °C for five minutes (Figure 10d).

## 6. Patents

A utility model for the StingReady device has been submitted and admitted for processing by the Spanish Patent and Trademark Office (OEPM), under application number U202530786, with a filing date of 30 April 2025. The invention is classified within the field of medicine, specifically allergology and immunotherapy, and relates to diagnostic and therapeutic methods for insect venom allergy.

## Figures and Tables

**Table 1:** Summary of patient data from the sting challenge test (SCT), including SCT date, local reaction, reaction grade (mild, moderate, or severe), and systemic reaction.

Patient	VIT Duration (in Months)	Specific IgE to Vespula (KU/L)	Local Reaction (in cm)	Systemic Reaction
1	44	3.33	10 × 9	no
2	62	6.56	7 × 6	no
3	63	1.79	8 × 5	no
4	7	23.4	1 × 1	yes
5	43	3.19	6 × 5	no
6	62	7.38	18 × 9	no
7	50	0.26	6 × 6	no
8	38	29.8	4 × 3	no
9	61	0.33	3 × 3	no

**Table 2:** Clinical characteristics of the patients who underwent a sting challenge test.

Patient	Sex	Age (in Years)	Culprit Insect (Species/No. of Stings)	Previous Stings by *V. velutina*	Grade of Anaphylaxis *	Total IgE (IU/mL)	Specific IgE to Vespula (KU/L)	VIT
1	male	44	*V. velutina*/1	No	1	408	7.0	*Vespula* spp.
2	male	42	*V. velutina*/1	No	1	86	4.56	*Vespula* spp.
3	male	69	*V. velutina*/2	No	1	1489	13.8	*Vespula* spp.
4 *	male	68	*V. velutina*/1	Yes	2	145	24.8	*Vespula* spp.
5 *	male	54	*V. velutina*/1	Yes	2	116	2.44	*Vespula* spp.
6	male	25	*V. velutina*/1	No	2	263	4.88	*Vespula* spp.
7	male	66	*V. velutina*/1	No	1	34	2.09	*Vespula* spp.
8	male	43	*V. velutina*/3	No	2	578	63.3	*Vespula* spp.
9	female	66	*V. velutina*/1	No	1	19	0.95	*Vespula* spp.

* According to [28].

## Data Availability

The original contributions presented in this study are included in the article/Supplementary Materials. Further inquiries can be directed to the corresponding author.

## Supplementary Materials

The following supporting information can be downloaded at https://www.mdpi.com/article/10.3390/toxins17060260/s1, Video S1: Demonstration of the entire *V. velutina* capture process at the bait station.

## Abbreviations

The following abbreviations are used in this manuscript:

CCh	Containment Chamber
GC	Glinding Component
SCT	Sting Challenge Test
VIT	Venom Inmmunotherapy
